# Targeting Platelet Thrombin Receptor Signaling to Prevent Thrombosis

**DOI:** 10.3390/ph6080915

**Published:** 2013-08-02

**Authors:** Eric L. Wallace, Susan S. Smyth

**Affiliations:** 1 Division of Cardiovascular Medicine and the Gill Heart Institute, University of Kentucky, Lexington, KY 40536, USA; 2 Lexington VA Medical Center, Lexington, KY 40536, USA

**Keywords:** platelets, thrombin, thrombosis, protease-activated receptors

## Abstract

Platelets contribute fundamentally to ischemic heart disease, and antiplatelet therapy has been critical to reducing acute thrombotic complications of atherosclerotic disease. Thrombin, by acting on protease activated receptors (PAR), is one of the most potent platelet activators. PAR-1 antagonists may therefore provide more comprehensive antithrombotic effects. We review the pathophysiology of atherothrombosis, platelet activation by thrombin, the role of platelet protease activated receptors (PAR), and the clinical data supporting their use.

## 1. Introduction

**Figure 1:**
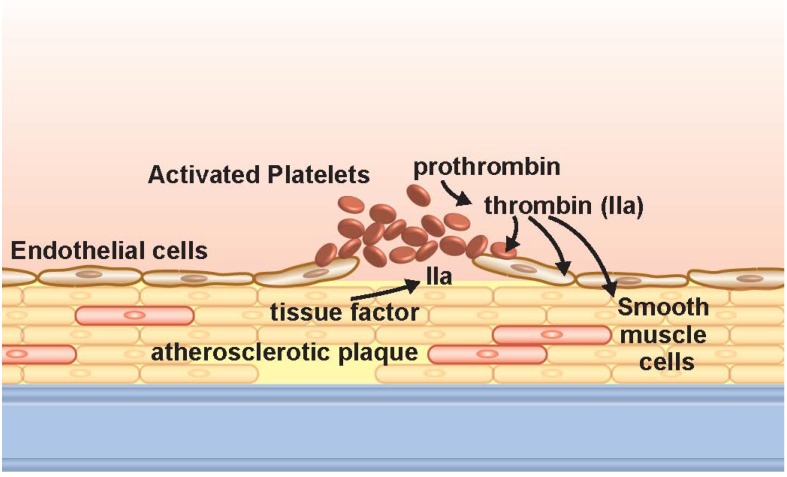
Platelet adherence to the endothelium occurs at the site of vascular injury, often in an area of atherosclerosis. Damage, erosion, or rupture of the endothelial surface, or an underlying atherosclerotic plaque exposes subendothelial matrix to which platelets adhere and are activated (see Figure 2). Tissue factor may also be present and result in the generation of thrombin (IIa). Additionally, thrombin can be generated along the surface of activated platelets or released microparticle (not depicted). Thrombin, in turn, can elicit effects on platelets, and endothelial or smooth muscle cells in the area.

Platelets play an essential role in cardiovascular disease and cardiac mortality. Their importance has been demonstrated in several landmark clinical trials in which inhibition of platelet activation and/or aggregation, improved cardiovascular outcomes [1,2]. Complex and intertwined systems regulate platelet function. Platelets can be activated by diverse soluble mediators, such as thromboxane A_2 _(TXA_2_) and adenosine diphosphate (ADP), adhesive interactions, and enzymes such as thrombin (Figure 1). Clinically, the most tractable pharmacologic targets for antiplatelet therapy have been signaling, mediated by the generation of TXA_2_ and by ADP acting on P2Y_12_ receptors, which are the targets for aspirin and P2Y_12_ antagonists, respectively. Indeed, both aspirin and P2Y_12_ antagonists are standard of care for acute coronary syndromes to prevent platelet thrombus formation following stent deployment, and in other settings.

Aspirin, which was developed more than 100 year ago, remains one of the most effective antiplatelet therapies. Early clinical trials demonstrated a significant absolute mortality benefit of aspirin in acute myocardial infarctions (AMI), that has yet to be duplicated in contemporary practice. More recently, targeting the P2Y_12_ receptor to blunt ADP signaling has demonstrated clinical efficacy [2,3,4]. Even in the face of drugs to limit TXA_2_ and ADP signaling, persistent platelet activation may contribute to recurrent events in the setting of AMI as well as unacceptably high rates of mortality [5,6]. Thus, alternant antiplatelet agents are still needed to reduce adverse cardiovascular outcomes.

To further improve outcomes, several newer agents target the platelet-activating effects of thrombin, one of the most potent stimuli for platelet activation [7]. This article will review the pathophysiology of atherothrombosis, platelet activation by thrombin, the role of platelet protease activated receptors (PAR), and clinical data for PAR antagonists.

**Figure 2:**
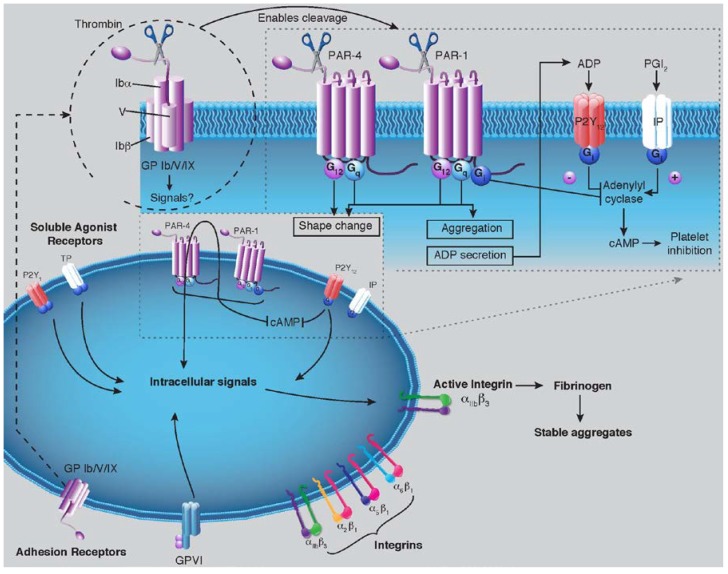
Platelet activation, aggregation and fibrin formation. Platelet activation, aggregation and fibrin production, occur via complex intracellular signaling processes and are regulated by cellular receptors. Platelets adhere to and then are activated by collagen, von Willebrand factor (vWF), and other adhesive proteins. These interactions are triggered when adhesive proteins interact with platelet glycoprotein (GP) receptors, several of which are members of the integrin family. Key GP receptors are displayed above. Platelet activation is reinforced with the release of contents, which includes ADP and other mediators. Thrombin can be generated locally or on the activated-platelet surface. Thrombin then cleaves to the protease-activating receptors (PAR) to expose a tethered ligand that serves to self-bind to PAR, triggering a confirmation that allow interactions with G proteins. PAR-1 couples to Gq protein to stimulate platelet activation. Additional, Gi-coupled pathways attenuate adenylyl cyclase activity, thereby lowering intracellular levels of the platelet inhibitor cAMP. PAR also couples to Gq proteins to stimulate platelet activation. The major platelet receptor GPIIb/IIIa (integrin αIIbβ3), mediates the final common pathway of platelet activation by undergoing a conformational change that enables the binding of multivalent ligands, such as fibrinogen and vWF, which cross-link adjacent platelets into aggregates.

## 2. Discussion

### 2.1. Platelet Biology and Thrombin

Platelets, small cell fragments, normally circulate in a resting state. Their main function is to initiate the process of clot formation. Tight regulation of the activate state of platelets allows them to react to vascular injury while preventing unnecessary thrombosis. Once vascular damage occurs, platelets encounter the connective tissue matrix underlying normal endothelial cells. This exposure results in their binding to collagen, von Willebrand factor (vWF) and other adhesive proteins via the platelet glycoprotein (GP) receptors GPVI, integrin α2β1 (GP Ia/IIa), GP Ib/IX, and integrin αIIbβ3 (GP IIb/IIIa). Binding of platelets to adhesion proteins begins the process of platelet activation and leads to a cascade in which platelets secrete granule contents that are rich in soluble mediators such as ADP and serotonin; arachidonic acid is metabolized to TXA_2_, and thrombin is generated along the growing thrombotic surface. As a consequence of granule cargo release and TXA_2_ and thrombin generation, surrounding platelets are activated. During the process of secretion, as granules fuse with the plasma membrane, P-selectin is exposed on the activated surface, and together with soluble mediators generated and released by platelets P-selectin recruits leukocytes to the platelet thrombus. The platelet aggregate, held together with fibrinogen and tethered with leukocytes, serves as a surface for production of additional thrombin (Figure 2).

In *ex vivo* systems, thrombin is one of the most potent activators of platelets known [7,8]. Thrombin also promotes the activation of coagulation factors V, VIII, XI, and XIII and catalyzes the conversion of fibrinogen into fibrin, and thus is indispensable for fibrin clot formation. The absence of prothrombin results in fatal perinatal lethality in mice, a phenotype that can be recapitulated by the combined deficiency in fibrinogen and the major platelet thrombin receptor, but not either alone [9].

In addition to playing a central role in hemostasis and thrombosis, platelets and thrombin may also serve as mediators of atherosclerosis. Systemic inflammation can lead to heightened expression of cell-adhesion molecules and binding of platelets [10]. Once adhered, platelets also secrete atherogenic mediators, such as cytokines, chemokines, growth factors, adhesion molecules, and coagulation factors to interact with leukocytes. Once upregulated by inflammation, there is growing evidence that these complex platelet-leukocyte interactions produce further aggregates that may play a central role in plaque formation and progression [11]. The evolving interplay between coagulation and inflammation in atherosclerosis may provide an emerging role for thrombin-specific inhibitors.

### 2.2. PAR-1 Structure and Mechanisms

The hormonelike actions of thrombin on cells are mediated by the protease activated receptor (PAR) family of G-protein coupled receptors (GPCR), which has four family members, PAR-1 to -4. Proteases activate signaling by cleaving the PAR receptor to expose a tethered ligand that is actually part of the receptor complex itself [12,13]. The tethered ligand binds the N-terminal transmembrane portion of the PAR; the intracellular C-terminus activates intracellular signaling [14]. Unlike other GPCRs, receptor activation by proteolysis is irreversible and terminated by receptor degradation.

PAR-1, involved in the initial response of human platelets to thrombin, responds to subnanomolar concentrations, whereas PAR-4 requires substantially higher concentrations of thrombin and appears to sustain platelet activation [13,15]. While PAR-2 and PAR-3 do not contribute to human platelet functions (although PAR-3 serves as a cofactor for thrombin binding on mouse platelets), the PAR receptors are widely expressed and PAR-1—and the other PAR receptors—contribute to various endothelial and vascular smooth muscle cell function [14].

Mice lacking the major thrombin receptor do not exhibit spontaneous bleeding, whereas mice (and humans) lacking major adhesive receptors GPIIb/IIIa or GPIb/IX, suffer from a bleeding diathesis. These and other observations led to speculation that thrombin signaling may contribute to thrombosis selectively over hemostasis, especially when compared to other anti-platelet targets such as the P2Y_12_ receptor) [16]. If correct, then drugs targeting thrombin platelet signaling might be expected to prevent thrombotic events without provoking excessive bleeding [13,17,18]. The hypothesis was supported by preclinical trials in monkeys, where no bleeding risk was observed when a PAR-1 inhibitor (SCH 530348 at 1 mg/kg) was administered either alone or in combination with aspirin plus clopidogrel [19]. Several large-scale clinical trials were undertaken to test the premise.

### 2.3. Clinical Trials Involving PAR-1

Two Thrombin Receptor Antagonists (TRA) that inhibit PAR-1 have been studied in clinical trials: SCH 530348 and E-5555. The results are summarized below. 

#### 2.3.1. Vorapaxar (SCH 530348)

Vorapaxar (Merck-Schering Plough; SCH 530248) is a selective high-affinity, orally active competitive PAR-1 antagonist. The drug undergoes oxidative metabolism through the CYP3A4 enzymes, is 90% excreted in bile, and has a half-life ranging from ~5 to 11 days. In phase I clinical trials, as a single high dose (20–40 mg) vorapaxar was potent, fast acting, and resulted in prolonged inhibition of Thrombin Receptor Activating Peptide (TRAP)- induced platelet aggregation (>80% inhibition at 1 h that was sustained for >72 h) [20]. A daily dose of vorapaxar 2.5 mg sustained the inhibitory effect for 28 days. The irreversibility of vorapaxar is attributed to a very slow dissociation rate from PAR1. The recently solved crystal structure of PAR1 revealed that vorapaxar bind close to the extracellular surface. Molecular dynamic simulations suggest that vorapaxar may stabilize an inactive conformation of the receptor [21].

The safety of vorapaxar was examined in several phase II clinical studies. Thrombin Receptor Antagonist in Percutaneous Coronary Intervention (TRA-PCI) was a multicenter, double-blinded randomized control trial of 1,030 patients referred to undergo cardiac catheterization, or a non-urgent PCI. Five hundred and seventy-three individuals were randomized in a 3:1 ratio to a loading dose of SCH 530348 (10 mg, 20 mg, or 40 mg), or matching placebo. Those in the SCH 530348 group who underwent PCI, continued taking an oral maintenance dose (0.5 mg, 1.0 mg, or 2.5 mg per day), and patients in the placebo group continued placebo for 60 days. The SCH 530348 dose-dependently inhibited TRAP-induced platelet aggregation, with the 40 mg dose demonstrating >90% inhibition at 2 h that was sustained by both the 1.0 mg and 2.5 mg daily maintenance doses. There was no difference in the primary safety endpoint, which was incidence of clinically significant major or minor bleeding, according to the thrombolysis in myocardial infarction (TIMI) scale (2.8% *vs.* 3.3%; in those taking SCH 530348 versus those with placebo, respectively; *p* = 0.77). A secondary efficacy endpoint of 60-day death or major adverse cardiovascular events (MACE), although not statistically significant, was reduced by SCH 530348 (5.9% *vs.* 8.6%) [22].

The Non-ST-segment elevation ACS (NSTE-ACS) study out of Japan randomized 117 patients to receive a 20 mg loading dose, 40 mg, or placebo in a 1:1:1 fashion. Patients receiving a loading dose were randomized to receive a 1.0 mg or 2.5 mg daily maintenance dose, in addition to standard care therapy of ASA and a P2Y12 antagonist. The incidence of TIMI major bleeding was 14% in the SCH 530348 arm, and 10% in placebo (point estimate of the difference of 4.6%; CI, 10.4–19.5). The secondary endpoint of periprocedural MI or recurrent non-fatal MI, was significantly reduced in patients taking SCH 530348 (16.9% *vs.* 42.9%; *p* = 0.013) [23].

Combined, these two phase II trials supported the notion that vorapaxar may provide cardiovascular benefit in patients undergoing percutaneous revascularization, in addition to standard dual-platelet inhibition with little bleeding risk. Two phase III trials quickly followed.

The Thrombin Receptor Antagonist for Clinical Event Reduction in Acute Coronary Syndrome (TRACER) trial was the first published phase III clinical trial to test PAR-1 inhibition to improve cardiovascular outcomes. TRACER was designed as a multinational, double-blind, randomized trial with a goal enrollment of 20,000 high-risk, non-ST-elevation MI (NSTEMI) patients. Participants were randomized in a 1:1 fashion to vorapaxar (40 mg loading dose and 2.5 mg daily), or placebo on top of standard medical therapy. The primary end point was a composite of death from cardiovascular causes, myocardial infarction, stroke, recurrent ischemia with re-hospitalization, or urgent coronary revascularization. In January 2011, a safety review board review revealed more bleeding events in the vorapaxar group, which led to trial termination prior to meeting the pre-specified number of primary efficacy events. At the time of the review, 12,944 subjects were enrolled. After a median follow-up of 502 days, the 2-year rate of MACE was lower in the vorapaxar than the placebo arm (18.5% *vs.* 19.9%), but was not statistically significant (hazard ratio [HR], 0.92; 95% CI, 0.85–1.01; *p* = 0.07). An important efficacy secondary endpoint composite of death from cardiovascular causes, myocardial infarction, or stroke also favored vorapaxar, (14.7% and 16.4%; HR, 0.89; 95% CI, 0.81–0.98; *p* = 0.02). However, rates of moderate and severe Global Utilization of Streptokinase and t-PA for Occluded Coronary Arteries (GUSTO) bleeding were 7.2% in the vorapaxar group, and 5.2% in the placebo group (HR, 1.35; 95% CI, 1.16–1.58; *p* < 0.001). Importantly, intracranial hemorrhage rates were 1.1% and 0.2%, respectively (HR, 3.39; 95% CI, 1.78–6.45; *p* < 0.001). The excess bleeding events with vorapaxar continued to increase during patient follow-up. This led the authors to conclude that the two-year duration of therapy may have affected the risk/benefit profile since dual-antiplatelet benefit is not seen beyond one year [24].

The use of vorapaxar was also studied in patients with stable atherosclerotic disease in the Thrombin Receptor Antagonist in secondary prevention of atherothrombic ischemic events (TRA2P-TIMI-50) trial. TRA2P-TIMI 50 was a multicenter, double-blind randomized control trial of 26,499 patients with a history of coronary artery disease, cerebrovascular disease, or peripheral vascular disease. The primary efficacy end point was the composite of death from cardiovascular causes, myocardial infarction, or stroke in patients treated with 2.5 mg of vorapaxar daily for 36 months. Bleeding outcomes were assessed by GUSTO definitions. Similar to the TRACER study, which was stopped early due to excess bleeding in January 2011, a press release announced the decision to discontinue the study drug in patients who had a history of stroke or experienced one during the course of the study. At 3-year follow up of the total population, the primary efficacy end-point had occurred in 9.3% in the vorapaxar group and 10.5% in the placebo group (HR, 0.87; 95% CI, 0.80–0.94; *p* < 0.001). The major safety end point of moderate or severe bleeding occurred in 4.2% in the vorapaxar group, as compared to 2.5% in the placebo group (HR, 1.66; 95% CI, 1.43–1.93; *p* < 0.001). Intracranial hemorrhage rates were higher in the patients receiving vorapaxar (1.0% *vs.* 0.5%, HR 1.94; 95% CI, 1.39–2.70; *p* < 0.001). In the subgroups without a history of stroke, the rates of intracranial hemorrhage were lower (0.6% in the vorapaxar group and 0.4% in the placebo group, *p* = 0.049) [25]. Post-hoc analysis of TRA2P-TIMI 50 indicated that vorapaxar reduced acute-limb ischemia and peripheral revascularizations in patients with peripheral arterial disease [26].

#### 2.3.2. Atopaxar (E5555)

The second oral PAR-1 inhibitor evaluated in clinical studies was atopaxar (E5555; Eisai, Tokyo, Japan). Similar to vorapaxar, atopaxar is a low molecular weight, orally active, hepatically-metabolized PAR-1 antagonist with demonstrated antiplatelet effects. *In vivo* studies demonstrate maximum inhibition of thrombin induced platelet aggregation at 6 h [27]. Effects on platelet function in blood samples were assessed on healthy subjects (n = 10), patients with coronary artery disease (CAD) treated with aspirin (n = 10), and patients with CAD treated with aspirin plus clopidogrel (n = 10). Thrombin receptor-activating, peptide-induced platelet aggregation was inhibited almost completely at all tested doses of E-5555 (20 and 50 ng/mL and 100 mg/mL), without dose-dependent effect [28].

The Japanese-Lessons from Antagonizing the Cellular Effect of Thrombin (J-LANCELOT) was the first clinical trial published on Atopaxar. The trial was actually two multicenter, randomized, double-blind, placebo-controlled phase II studies to assess the safety and efficacy of E5555, in addition to standard therapy in Japanese patients with acute coronary syndrome (ACS) or high-risk coronary artery disease (CAD). Patients with ACS (n = 241) or high-risk CAD (n = 263) received E5555 or placebo in a 3:1 fashion. Subjects randomized to receive the drug were assigned in a 1:1:1 manner to three daily doses (50, 100, or 200 mg) for 12 weeks (ACS), or 24 weeks (CAD patients). Thrombin receptor-activating peptide (TRAP)-induced platelet aggregation was assessed at sites with capable facilities. Daily doses of 100 and 200 mg in CAD and ACS subjects resulted in mean platelet aggregation inhibition of >90%; whereas, the 50 mg daily dose of E5555 reduced aggregation to 20–60%. The primary outcome was safety as assessed by bleeding events using both CURE (Clopidogrel in Unstable Angina to Prevent Recurrent Events) and TIMI definitions. In the ACS population, bleeding rates were numerically lower in patients receiving placebo compared to E5555 (16.4% *vs.* 19.4%, *p* = 0.61), but neither group had any TIMI major bleeding events. Similarly, in the stable CAD group, any TIMI bleeding was lower but not statically significant (4.5% *vs.* 9.6%, *p* = 0.21). Again, no TIMI major bleeding was observed in either group. Rates of CURE bleeding were also not significantly different in the E5555 or placebo groups for ACS or CAD patients. The rate of major cardiovascular adverse events in the combined E5555 group was not different from placebo in ACS patients (6.6% placebo *vs.* 5.0% E5555, *p* = 0.73) or CAD (4.5% placebo *vs.* 1.0% E5555, *p* = 0.066). There was a statistically significant dose-dependent increase in liver function abnormalities (AST or ALT > 3 times the upper limit of normal) and QTc prolongation reported [29].

Results of the Lessons from Antagonizing the Cellular Effect of Thrombin—Acute Coronary Syndrome (LANCELOT-ACS) and Lessons from Antagonizing the Cellular Effect of Thrombin—Coronary Artery Disease (LANCELOT-CAD) studies were published one year later. Both trials were similar to the phase II J-LANCELOT study in design but larger in recruitment. LANCELOT–ACS enrolled 603 patients within 72 h of ACS presentation, and subjects were randomized 1:1:1:1 to treatment with one of three dosing levels of atopaxar (400-mg loading dose followed by 50, 100, or 200 mg daily) or matching placebo in addition to standard therapy with ASA and P2Y_12_ antagonist. The primary safety endpoint was the proportion of subjects with major bleeding according to the CURE and TIMI bleeding classification. Secondary objectives included the effects of atopaxar on major adverse cardiovascular events, including cardiovascular death, MI, stroke, or recurrent ischemia, in addition to platelet function analysis. The incidence of CURE major bleeding was numerically higher but not statistically significant in the atopaxar group, compared with the placebo group (1.8% *vs.* 0%; *p* = 0.12), and the combined endpoint of major or minor bleeding was also similar (3.1% versus 2.2%, *p =* 0.63). No dose-related trend was seen (*p* = 0.80). Similarly, there were no differences observed by TIMI major bleeding definition (0% *vs.* 1.3%) slightly favoring placebo. Secondary outcomes of MACE was also similar between the atopaxar and placebo arms (8.0% versus 7.8%; *p* = 0.93). A transient dose-dependent rise in transaminase levels was observed, but none required termination of treatment and all resolved by the end of the study follow up [30].

Published simultaneously was the LANCELOT-CAD study, which randomized 720 patients with high-risk CAD. High risk was defined as previous ACS or MI at least four weeks previously, percutaneous coronary revascularization at least 12 weeks previously, or angina with documented ischemia and high risk features. Patients were again randomized in a 1:1:1:1 manner to atopaxar (50 mg, 100 mg, or 200 mg daily) *vs.* placebo. The primary outcome was any TIMI or CURE bleeding through the 24-week study duration. The secondary endpoint was efficacy as determined by MACE, which was the composite of cardiovascular death, myocardial infarction, stroke, or refractory ischemia. Overall CURE bleeding was significantly higher in patients receiving atopaxar than placebo (3.9% *vs.* 0.6%, RR, 6.8, *p* = 0.03), and similar to TIMI bleeding as well (10.3% *vs.* 6.8%, RR 1.5, *p* = 0.17, respectively). There were no differences in major bleeding seen. The MACE secondary endpoint was numerically lower for those receiving atopaxar (2.6% *vs.* 4.6%), but not by a significant margin (*p* = 0.77). Similar to the other atopaxar trials, transient transaminase elevations were noted. Additionally, modest QTc changes were seen, but prolongations > 30 milliseconds were infrequent and not significantly different between the two groups [31].

### 2.4. Pooled Analysis of Current Trials and Major Bleeding

A meta-analysis was recently published compiling all the data from the vorapaxar and atopaxar studies. A total of 42,355 subjects met inclusion criteria from seven published reports and no publication bias was noted by the authors. In their analysis, PAR-1 antagonists were associated with a statistically non-significant, numerically lower risk of cardiovascular mortality than that seen with placebo (RR, 0.93; 95% CI, 0.83–1.04; *p* = 0.20). Key secondary outcomes concluded that PAR-1 antagonists were significantly associated with a lower risk of recurrent MI; (RR, 0.78; 95% CI, 0.67–0.92; p = 0.003) but greater incidence of TIMI major bleeding; RR 1.46 (95% CI, 1.29, 1.65, *p* < 0.0001) [32].

The higher rates of adverse bleeding events with PAR-1 antagonists, specifically intracranial hemorrhage, remain a significant concern for clinical practice. Retrospective analysis of TRA2P-TIMI 50 has implicated pre-existing stroke as a significant predictor for intracranial hemorrhage (0.2% *vs.* 0.8% per year) [25]. These findings are not unique to this class of antithrombotic therapy. Previous clinical trials with more potent platelet inhibition with P2Y_12_ therapy have also found elevated risk of severe intracranial bleeding in patients with pre-existing stroke [4,33,34]. Balancing adverse events remains a challenge in this high-risk population where future cardiovascular events also occur more frequently than those without pre-existing stroke. In fact in TRA2P, despite major bleeding concerns, the overall net clinical benefit of cardiovascular death, myocardial infarction, or stroke (for those with any history of stroke), did not show overall harm and actually had numerically lower event rates (15.2% *vs.* 16.4%, HR 0.95, CI, 0.80, 1.11, *p* = 0.50). Thus, a treatment strategy assessing individual bleeding risk would likely be necessary to balance the possible benefits. Assessment for known intracranial bleeding risk factors such as hypertension, alcohol consumption, cerebral microbleeds, white matter hyperintensities, and common genetic variants, may be helpful while at the same time attempting to further reduce cardiovascular events in this high-risk population [35]. Although these risk factors represent a potential tool to lower cardiovascular risk in those with an acceptable risk profile, further studies are warranted to examine the accuracy of these and other clinical models in the PAR-1 study populations.

### 2.5. Future Pharmacological Agents

Currently there are several other selective PAR-1 antagonists, SCH 205831 and SCH 602539, undergoing early clinical evaluation. Both have been studied in animal models and appear to be potent inhibitors of thrombosis in a dose-dependent manner, similar to vorapaxar and atopaxar. Further clinical studies are likely to be forthcoming [36]. Additionally, thrombin receptor-signaling has been targeted by novel cell-permeant peptide, PZ-128, which targets the interactions between PAR1. In guinea pigs and non-human primates, PZ-128 synergized with clopidogrel to inhibit thrombosis, without a prolongation of bleeding [37].

### 2.6. PAR-1 Inhibitors and P2Y_12_ Antagonism

After preclinical studies of PAR-1 inhibition did not demonstrate an increased bleeding risk when administered in combination with aspirin plus clopidogrel, there was speculation that PAR-1 inhibition might prevent thrombotic events, without provoking excessive bleeding. This initial excitement has been blunted by the results of the vorapaxar and atopaxar clinical trials, where the net clinical benefit of thrombin inhibition has been offset by excess of major bleeding events. Questions remain regarding the general applicability of these findings to all populations, or whether or not certain subgroups exist that may demonstrate cardiovascular benefit without bleeding risk.

In TRACER, there was enhanced clinical efficacy with vorapaxar compared to placebo for both the primary (HR, 0.77; 95% CI 0.60–0.99) and secondary end points (HR, 0.74; 95% CI 0.57–0.97), in patients who were not treated with thienopyridine at randomization. In fact, in patients who did not receive P2Y_12_ inhibition but had PAR-1 inhibition, lower rates of the combined clinical outcome actually compared with those who received thienopyridine therapy (18.3% *vs.* 19.3%). The hazard of GUSTO was that moderate or severe bleeding in the vorapaxar group, was also lower in patients who were not receiving a thienopyridine at randomization, whereas the risk was increased in patients who were receiving a thienopyridine (HR, 0.95; 95% CI, 0.65–1.40 with no thienopyridine; HR, 1.45; 95% CI, 1.23–1.71 with thienopyridine; *p* = 0.04 for interaction [25]). 

The implications of these findings are unknown and further studies are needed to better understand the interplay between PAR-1 and P2Y_12_ inhibition, and what populations might be best served by more potent antithrombotic therapy. On the one hand, PAR-1 therapy may serve as an alternative to P2Y_12_ inhibitors in patients who are either non-responders or resistant. Possibly these observations will trigger future studies that will directly compare PAR-1 blockade with P2Y_12_ inhibition.

### 2.7. Comparison with New Oral Anticoagulants

Factor Xa inhibitors (apixaban, rivoraxaban) and direct thrombin inhibition (dabigitran), serve as newer therapeutic targets offering comparable efficacy to warfarin for both the reduction of stroke in atrial fibrillation and rates of recurrent venous thromboembolism in deep venous thrombosis [38,39,40,41,42]. Interestingly, in comparison to warfarin, all three of the new agents have comparable or lower rates of ICH, but a tendency to elicit gastrointestinal bleeding (highest for dabigatran and lowest for apixaban).

In the setting of acute coronary syndromes, rivoraxaban (2.5 mg twice daily, a quarter of the dose used for stroke prevention in atrial fibrillation) reduced a composite endpoint of death from cardiovascular causes, myocardial infarction, or stroke and demonstrated a survival benefit when added to standard of care (that largely included aspirin and P2Y12 antagonism) [43]. The survival benefit was not observed with higher dose (5 mg twice daily); similar to PAR-1 antagonists, both doses increased the rate of intracranial bleeding. On the other hand, dabigatran has been associated with higher rates of MI in several analyses of data from non-ACS trials [38,41]. Thus, there may be differences in clinical benefit of upstream (FXa), direct, and downstream (PAR-1) thrombin inhibition. Whether the observed differences reflect underlying biology of hemostasis and thrombosis, patient selection, dosing, random chance or other process, is not known. 

## 3. Conclusions

Platelet inhibition has demonstrated significant benefit in the treatment of ischemic heart disease but residual mortality remains. The development of newer and novel antiplatelet therapies to combat thrombosis is welcomed. PAR-1 antagonists may provide more comprehensive antithrombotic effects with the potential to reduce atherothrombotic complications. To date, clinical trials have assessed PAR-1 inhibition on top of standard of care, which included aspirin and often, P2Y_12_ antagonism. In this setting, increased bleeding events have been noted. Whether PAR1 inhibitors will serve in a complimentary manner or replace any current therapy, remains unknown. Retrospective analysis of the TRACER trial did suggest lower bleeding in patients not also taking a P2Y_12_ antagonist. The results of ongoing clinical trials will hopefully provide the background for further large clinical trials and better determine how we will use these agents in the future.
